# Xiasangju alleviate metabolic syndrome by enhancing noradrenaline biosynthesis and activating brown adipose tissue

**DOI:** 10.3389/fphar.2024.1371929

**Published:** 2024-03-21

**Authors:** Changhao He, Yongcheng An, Lu Shi, Yan Huang, Huilin Zhang, Wanxin Fu, Menglu Wang, Ziyi Shan, Yuhang Du, Jiamei Xie, Zhiyun Huang, Weiguang Sun, Yonghua Zhao, Baosheng Zhao

**Affiliations:** ^1^ Department of Pharmacology of Chinese Materia Medica, School of Chinese Materia Medica, Beijing University of Chinese Medicine, Beijing, China; ^2^ Central Laboratories, Qingdao Municipal Hospital, University of Health and Rehabilitation Sciences, Qingdao, China; ^3^ College of Life Sciences, Beijing University of Chinese Medicine, Beijing, China; ^4^ Guangzhou Baiyunshan Xingqun Pharmaceutical Co., Ltd, Guangzhou, China; ^5^ State Key Laboratory of Quality Research in Chinese Medicine, Institute of Chinese Medical Sciences, University of Macau, Taipa, China; ^6^ Beijing Research Institute of Chinese Medicine, Beijing University of Chinese Medicine, Beijing, China

**Keywords:** Xiasangju, metabolic syndrome, adipose tissue, noradrenaline biosynthesis, gut microbiome

## Abstract

Metabolic syndrome (MetS) is a clinical condition associated with multiple metabolic risk factors leading to type 2 diabetes mellitus and other metabolic diseases. Recent evidence suggests that modulating adipose tissue to adaptive thermogenesis may offer therapeutic potential for MetS. Xiasangju (XSJ) is a marketed drug and dietary supplement used for the treatment of metabolic disease with anti-inflammatory activity. This study investigated the therapeutic effects of XSJ and the underlying mechanisms affecting the activation of brown adipose tissue (BAT) in MetS. The results revealed that XSJ ameliorated MetS by enhancing glucose and lipid metabolism, leading to reduced body weight and abdominal circumference, decreased adipose tissue and liver index, and improved blood glucose tolerance. XSJ administration stimulated catecholamine biosynthesis, increasing noradrenaline (NA) levels and activating NA-mediated proteins in BAT. Thus, BAT enhanced thermogenesis and oxidative phosphorylation (OXPHOS). Moreover, XSJ induced changes in gut microbiota composition, with an increase in *Oscillibacter* abundance and a decrease in *Bilophila*, *Candidatus Stoquefichus*, *Holdemania*, *Parasutterella* and *Rothia*. XSJ upregulated the proteins associated with intestinal tight junctions corresponding with lower serum lipopolysaccharide (LPS), tumor necrosis factor *α* (TNF-*α*) monocyte chemoattractant protein-1 (MCP-1) and interleukin-6 (IL-6) levels to maintain NA signaling transport. In summary, XSJ may alleviate MetS by promoting thermogenesis in BAT to ultimately boost energy metabolism through increasing NA biosynthesis, strengthening intestinal barrier integrity and reducing low-grade inflammation. These findings suggest XSJ has potential as a natural therapeutic agent for the treatment of MetS.

## 1 Introduction

Metabolic syndrome (MetS) constitutes a clinical syndrome characterized by a constellation of interconnected metabolic risk factors, including obesity, hyperlipidemia, hyperglycemia, and hypertension. Central obesity and insulin resistance serve as core components of MetS (Alberti et al., 2009). Notwithstanding certain adverse effects, statins and thiazolidinediones (TZDs) are still effective drugs for treating MetS (Suckling, 2007). With the gradual increase in the prevalence of MetS internationally (Saklayen, 2018), there is a growing demand for the development of novel therapeutic interventions.

In recent years, adipose tissue has become a focus research area owing to its pivotal role in metabolism and endocrine. White adipose tissue (WAT) serves as an energy storage site, while in contrast, brown adipose tissue (BAT) is responsible for energy expenditure. BAT achieves this through the activation of uncoupling protein 1 (UCP1) to generate heat, which is called adaptive thermogenesis (Chouchani and Kajimura, 2019). BAT necessitates substantial glucose and fatty acid consumption through oxidative phosphorylation (OXPHOS) for thermogenesis, contributing to heightened energy expenditure (Chouchani and Kajimura, 2019; Park et al., 2023). Noradrenaline (NA, i.e., norepinephrine), one of the well-known thermogenic activators from the catecholamine family, promotes thermogenesis by beta-adrenergic receptors (*β*-ARs) on the brown adipocyte surface (Kajimura et al., 2015). Therefore, the activation of BAT presents a potential therapeutic strategy for obesity and metabolic disorders.

Gut microbiota-host interactions have become a subject of increasing interest. The progression of MetS has been demonstrated to correlate with gut microbiota (Ussar et al., 2015). The gut microbiota influences the progression of metabolic diseases directly through metabolism-dependent processes that involve the alteration of host metabolism. This includes the regulation of aromatic amino acids (AAAs). Additionally, there are metabolism-independent processes such as activation of inflammation by disruption of the intestinal barrier (Tang et al., 2017). Obesity is associated with systemic inflammation caused by increased intestinal barrier permeability (Acciarino et al., 2023). It seems to be a feasible approach to modulate MetS by intervening in the gut microbiota and intestinal barrier.

Xiasangju (XSJ) is a Chinese patent medicine derived from the classic formula *Sang Ju Yin* (*桑菊饮*) and also have been refined into XSJ herbal tea and XSJ beverage for dietary supplements. XSJ is composed of *Prunellae Spica* (*Prunella vulgaris* L.), mulberry (*Morus alba* L.) leaf and *Flos Chrysanthemi Indici* (*Chrysanthemum indicum* L.) (Table 1). The plant names have been verified using http://mpns.kew.org. XSJ is intended for the anti-inflammatory treatment of respiratory and liver diseases and has shown potential pharmacological effects in antioxidant, antidiabetic, and immunomodulatory areas (Wu et al., 2022). XSJ has been considered for metabolic disease treatment and its herbal components also have shown good effects in the reduction of blood glucose, lipid levels, and blood pressure (Peng, 2020). As previous review by S. Wu et al., XSJ undergoes a complete process of extraction, purification, and quality control. A series of studies use HPLC methods to determine the contents of chlorogenic acid, salviaflaside, rosmarinic acid, and linarin for XSJ quality control (Wu et al., 2022). However, unclear therapeutic effects and pharmacological mechanisms have hindered the widespread use of XSJ in clinical practice. Based on the cumulative research findings, we further explored the mechanism underlying the XSJ improvement in response to the systemic metabolic disorder of MetS through BAT activation.

**TABLE 1:** Composition of the formula for XSJ.

Herb	Chinese name	Latin name	Family	Part used	Occupied percent (%)
*Prunella vulgaris* L	Xia Ku Cao	*Spica Prunellae*	*Lamiaceae*	Fruitspike	66.22
*Chrysanthemum indicum* L	Ye Ju Hua	*Flos Chrysanthemi Indici*	*Asteraceae*	Capitulum	10.60
*Morus alba* L	Sang Ye	*Folium Mori*	*Moraceae*	Leaf	23.18

To elucidate the potential mechanism through which XSJ alleviates MetS, this study employed XSJ extract to investigate its regulatory effects on energy metabolism. The study systematically explored the gut microbiota and metabolite alterations caused by a high-fat, high fructose diet-induced MetS through 16S ribosomal DNA (rDNA)-based microbiota and metabolomics analysis of cecum contents. The principal outcomes of this study indicate that XSJ extract reduces lipid accumulation and increases lipid oxidation. XSJ may promote NA biosynthesis, activate BAT, restore intestinal barrier integrity, reduce inflammation, and thus enhance energy metabolism. In brief, XSJ holds potential as a natural therapeutic agent for the treatment of MetS.

## 2 Materials and methods

### 2.1 Animals and groups

Sixty specific-pathogen-free (SPF) male Sprague-Dawley rats (batch number:110324211103957112), 8-week-old and weighing (220 ± 20) g, were obtained from SiPeiFu Biotechnology Co., Ltd. (Beijing, China). The rats had *ad libitum* access to food and water, and were housed in the barrier environmental facility at Beijing University of Chinese Medicine, which was maintained at (23 ± 2) °C temperature, 55%–65% relative humidity, and a 12 h/12 h light/dark cycle. The experiment was approved by the Medical and Laboratory Animal Ethics Committee of Beijing University of Chinese Medicine and the Animal Ethics Committee of University of Macau, and the approval numbers were BUCM-4-2021071207-3178 and UMARE-008-2021.

Rats were acclimated for 1 week, then were randomly assigned 10 to the Normal group and 50 to the high-fat, high-fructose diet (Model) group based on body weight and fasting blood glucose (FBG) levels. During the 12-week modeling period, the rats in the Normal group were fed a standard chow diet (SWS9102), while those in the Model group were fed a high-fructose, high-fat diet (XT301-1). The diets were supplied by Xietong Pharmaceutical Bio-engineering Co., Ltd. (Jiangsu, China). And the specific compositions of these diets are shown in Supplementary Table S1. The evaluation of the MetS model was conducted according to a translational guide developed by Inês Preguiça (Preguiça et al., 2020). The evaluation criteria for MetS model in rats was determined by the presence of at least three out of the following five manifestations: 1) obesity; 2) reduced high-density lipoprotein; 3) elevated triglycerides; 4) elevated fasting blood glucose or impaired glucose tolerance; 5) elevated systolic blood pressure. Successful modeling is defined as having mean values in the Model group that are 10% higher than those in the Normal group.

### 2.2 Experimental procedures

The successful model rats were randomly divided into five groups (*n* = 8 per group) according to body weight, blood glucose and lipid levels: Model group, Rosiglitazone (Rosi) group, XSJ high dose (XSJH) group, XSJ medium dose (XSJM) group, and XSJ low dose (XSJL) group. The Normal and Model groups received equal amounts of distilled water daily.

The raw extract Xiasangju (XSJ) (Batch No. 20210812) were obtained from of the Chinese patent medicine Baiyunshan Xingqun XSJ granules (Chinese drug approval number Z44022217) provided by Baiyunshan Xingqun Pharmaceutical Co., Ltd. (Guangzhou, China). According to the conversion ratio between rat and humans, the daily extract dose (g/kg) of XSJ for rats can be calculated as the following formula: the conversion ratio of surface area between rat and human (6) × a daily dose of an adult (4.8 g extract)/the average weight of an adult (60 kg). That is: 6 × 4.8 (g)/60 (kg) = 0.48 g/kg as the rat equivalent dose. Therefore, in the study, 0.24 g/kg is regarded as low dose and 0.96 g/kg as high dose. And the rats of XSJ groups were given XSJ orally once daily. The rats of Rosi group were given rosiglitazone (No. H20030569, Hengrui, China) 0.4 mg/kg by gavage once a day as the daily human dose of rosiglitazone (4 mg/d).

During administration, the Normal group continued to be fed a standard chow diet and the other five groups were fed a high-fructose, high-fat diet. Body weight, abdominal circumference, nasoanal length and blood glucose were measured after a 12-h fast at a fixed time each week. And body mass index (BMI) was calculated as weight (kg)/[height (m)] ^ 2. Blood pressure was measured using the BP-2010A tail-cuff blood pressure measurement system (Softron Biotechnology, China) which records systolic (SBP), diastolic (DBP), and mean (MBP) arterial pressures with 3 repetitions at 15-min intervals.

### 2.3 Glucose tolerance test and homeostasis model assessment of IR index (HOMA-IR)

An oral glucose tolerance test (OGTT) was performed at the 12th week of high-fat, high-fructose diet modeling and the end of the 16th week of XSJ treatment. Before administering oral glucose loading (2.0 g/kg) for the OGTT, rats were fasted for 12 h. Blood glucose was measured before (0) and 15, 30, 60, 90, and 120 min after glucose administration with a glucometer (ACCU-CHEK^®^ Performa, Roche, U.S.A). Glucose levels were plotted over time and the area under the curve (AUC) of OGTT was calculated. HOMA-IR was calculated according to the following formula: fasting glucose levels (mmol/L) × fasting serum insulin (mU/L)/22.5.

### 2.4 Sample preparation

Rats were fasted for 10 h before each assay. After a 12-week modeling period, blood was collected from the retro-orbital venous plexus of rats after 4% isoflurane (RWD, China) inhalation anesthesia for model evaluation. After 16 weeks of XSJ administration, rats were anesthetized with 25% urethane (Yuanye, China) and blood was collected from the abdominal aorta. Serum was collected after centrifugation at 4°C, 3,000 rpm for 10 min. Samples of the liver, interscapular brown adipose tissue (BAT), epididymal white adipose tissue (eWAT), inguinal subcutaneous white adipose tissue (iWAT) and colon were collected. The organ coefficients for the liver, BAT, eWAT, and iWAT were calculated as follows: organ coefficient = organ weight (g)/rat body weight (kg) × 1000. Samples were either tested immediately after collection or frozen in liquid nitrogen and stored at −80°C.2.5.

### 2.5 Histological and transmission electron microscopy (TEM) staining

The pathology of liver, BAT, iWAT, and colon tissues was assessed using hematoxylin and eosin (H&E) staining. All tissue samples were fixed in 10% neutral formalin, dehydrated in ethanol, and embedded in paraffin wax. 5 μm-thick tissue sections were stained with H&E dye before pathology slice scanner observation (Roche, Switzerland). Hepatic fat accumulation was assessed using Oil Red O staining. Colon tissues were embedded in glutaraldehyde and dehydrated in an ethanol-acetone gradient, and colonic epithelial tight junctions were assessed by TEM.

### 2.6 Biochemical analysis

The biochemical assay kits were used to determine serum lipid profile (triglycerides (TG), total cholesterol (TC), high-density lipoprotein cholesterol (HDL-C) and low-density lipoprotein cholesterol (LDL-C)) in rats for model evaluation. The serum lipid indices, as well as the concentrations of TG and TC in both liver and BAT, were measured following a 16-week treatment period. All of the above-mentioned kits were acquired from Nanjing Jiancheng Bioengineering Institute, China. Lipopolysaccharide (LPS), tumor necrosis factor-alpha (TNF-*α*), monocyte chemotactic protein-1 (MCP-1), interleukin-6 (IL-6), glucagon-like peptide-1 (GLP-1) and insulin (INS) of rat serum were quantified by enzyme-linked immunosorbent assay (ELISA) kits (Kete, China).

### 2.7 Cecal content sample preparation and analysis for UHPLC-MS/MS system

100 mg of cecal contents were homogenized and resuspended in pre-chilled 80% methanol. After centrifuging at 15,000 g, 4°C for 20 min, then the supernatant was diluted to a final concentration of 53% methanol, transferred to a new tube and centrifuged again. Finally, the supernatant was injected into the UHPLC-MS/MS system (Thermo Fisher, Germany) using a 17 min linear gradient at a flow rate of 0.2 mL/min. The eluents for the positive polarity mode were eluent A (0.1% formic acid in Water) and eluent B (Methanol). The eluents for the negative polarity mode were eluent A (5 mM ammonium acetate, pH 9.0) and eluent B (Methanol). The solvent gradient was set as follows: 2% B, 1.5 min; 2%–85% B, 3 min; 85%–100% B, 10 min; 100%–2% B, 10.1 min; 2% B, 12 min. Mass spectrometer was operated in positive/negative polarity mode with spray voltage of 3.5 kV, capillary temperature of 320°C, sheath gas flow rate of 35 psi and aux gas flow rate of 10 L/min, S-lens RF level of 60, Aux gas heater temperature of 350°C. The raw data files generated by UHPLC-MS/MS were processed using Compound Discoverer 3.1 (CD3.1, Thermo Fisher, United States) to quantitation for each metabolite. These metabolites were annotated using the KEGG Database, HMDB Database and LIPIDMaps Database. Principal components analysis (PCA) and Partial least squares discriminant analysis (PLS-DA) were performed. We applied univariate analysis (*t*-test) to calculate the statistical significance (*p*-value). The metabolites with VIP >1, *p* < 0.05 and |log_2_(FoldChange)| ≥ 1.5 were considered to be differential. The metabolic pathways were enriched by differential metabolites using the KEGG Database.

### 2.8 16S rDNA sequence analysis

Total genomic DNA was extracted from 200 mg of cecal contents samples using the CTAB method and amplified with 341F—806R barcoded primers for the 16S rDNA V3—V4 regions. PCR products were mixed in equidensity ratios and purified with Qiagen Gel Extraction Kit (Qiagen, Germany). Then purified amplification products were sequenced to construct a sequencing library using TruSeq^®^ DNA PCR-Free Sample Preparation Kit (Illumina, United States). The sample library was sequenced on an Illumina NovaSeq platform. Sequences with ≥97% similarity were assigned to the same operational taxonomic units (OTUs) using the Silva database (http://www.arb-silva.de/) to annotate taxonomic information based on the Mothur algorithm.

According to the taxonomic classification and OTU abundance, the abundance of each sample at each taxonomic level of phylum, class, order, family and genus was calculated and visualized by phyloseq (McMurdie and Holmes, 2013). Macrogenomic function predictions were based on Tax4Fun2 (Wemheuer et al., 2020).

### 2.9 Mitochondrial DNA copy number

Total genomic DNA of BAT and iWAT was isolated by DNeasy Blood & Tissue Kit (Qiagen, United States) according to the manufacturer’s instructions. Quantitative PCR was carried out using a Step One Real-Time PCR System (Applied Biosystems, United States) and PCR products were detected by using the ChamQ Universal SYBR qPCR Master Mix (Vazyme, China). Mitochondrial DNA (mtDNA) copy number was estimated by quantifying the genomic DNA levels of mtDNA-transcribed *Cox2*, and normalized to that of the nuclear-specific gene *β*-actin (Kowluru et al., 2014). Primers used are listed in Supplementary Table S2.

### 2.10 Western blot analysis

The BAT and colon tissue were homogenized and the protein concentrations were measured using a BCA protein detection kit (NCMbiotech, China). Total protein (10 μg) was separated by SDS-PAGE, and transferred to PVDF membranes (Millipore, United States). Membranes were incubated overnight at 4°C with primary antibodies. BAT primary antibodies sources were as follows: Acyl-CoA oxidase 1 (ACOX1) (1:1000, ab184032, Abcam), Adipose triglyceride lipase (ATGL) (1:1000, ab207799, Abcam), Carnitine palmitoyl transferases 1 *α* (CPT1*α*) (1:1000, ab234111, Abcam), *β*3 Adrenoceptor (ADRB3) (1:1000, YT0363, Immunoway), Dopamine *β*-hydroxylase (DBH) (1:1000, YT1296, Immunoway), Protein kinase A (PKA) (1:10000, ab134901, abcam), Uncoupling Protein 1 (UCP1) (1:1000, 23673-1-AP, Proteintech), Peroxisome proliferator-activated receptor *α* (PPAR*α*) (1:1000, ab178860, Abcam), PPAR *γ* coactivator 1*α* (PGC-1*α*) (1:1000, ab188102, Abcam), PRD1-BF-1-RIZ1 homologous domain containing protein 16 (PRDM16) (1:1000, NBP1-71992, Novus), NADH dehydrogenase [ubiquinone] 1 beta subcomplex subunit 8 (NDUFB8) (1:5000, 14794-1-AP, Proteintech), Succinate dehydrogenase complex iron sulfur subunit B (SDHB) (1:5000, 10620-1-AP, Proteintech), Ubiquinol-cytochrome c reductase core protein 2 (UQCRC2) (1:2000, 14742-1-AP, Proteintech), Mitochondrially encoded cytochrome c oxidase II (MTCO2) (1:2000, 55070-1-AP, Proteintech), ATP synthase F1 subunit *α* (ATP5A1) (1:5000, 14676-1-AP, Proteintech) and *β*-Tubulin (1:1000, 10094-1-AP, Proteintech). Colon tissue primary antibodies sources were as follows: ZO-1 (1:1000, 21773-1-AP, Proteintech), Occludin (1:1000, 27260-1-AP, Proteintech), Claudin 1 (1:1000, 13050-1-AP, Proteintech) and *β*-Tubulin (1:1000, 10094-1-AP, Proteintech). Membranes were incubated for 60 min with Goat anti-Rabbit IgG-HRP (1:1000, ab205718, Abcam) at room temperature. Protein band signals were detected using the ChemiDoc MP Imaging System (Bio-Rad, United States). ImageJ software (NIH, United States) was used for quantitative analysis of protein bands. All target proteins were normalized by *β*-Tubulin.

### 2.11 Statistical analysis

GraphPad Prism 9.0 and R 4.3.1 were used to perform the statistical analyses and visualize the data. All data were expressed as the means ± standard deviation (SD). Statistical significance was tested with one-way ANOVA (and nonparametric or mixed). LSD test was used to assess intergroup differences for normal data. And Mann-Whitney U test was used to assess intergroup differences for non-normal data. *p* < 0.05 was considered statistically significant.

## 3 Results

### 3.1 High-fructose, high-fat diet leads rats to metabolic syndrome

**FIGURE 1:**
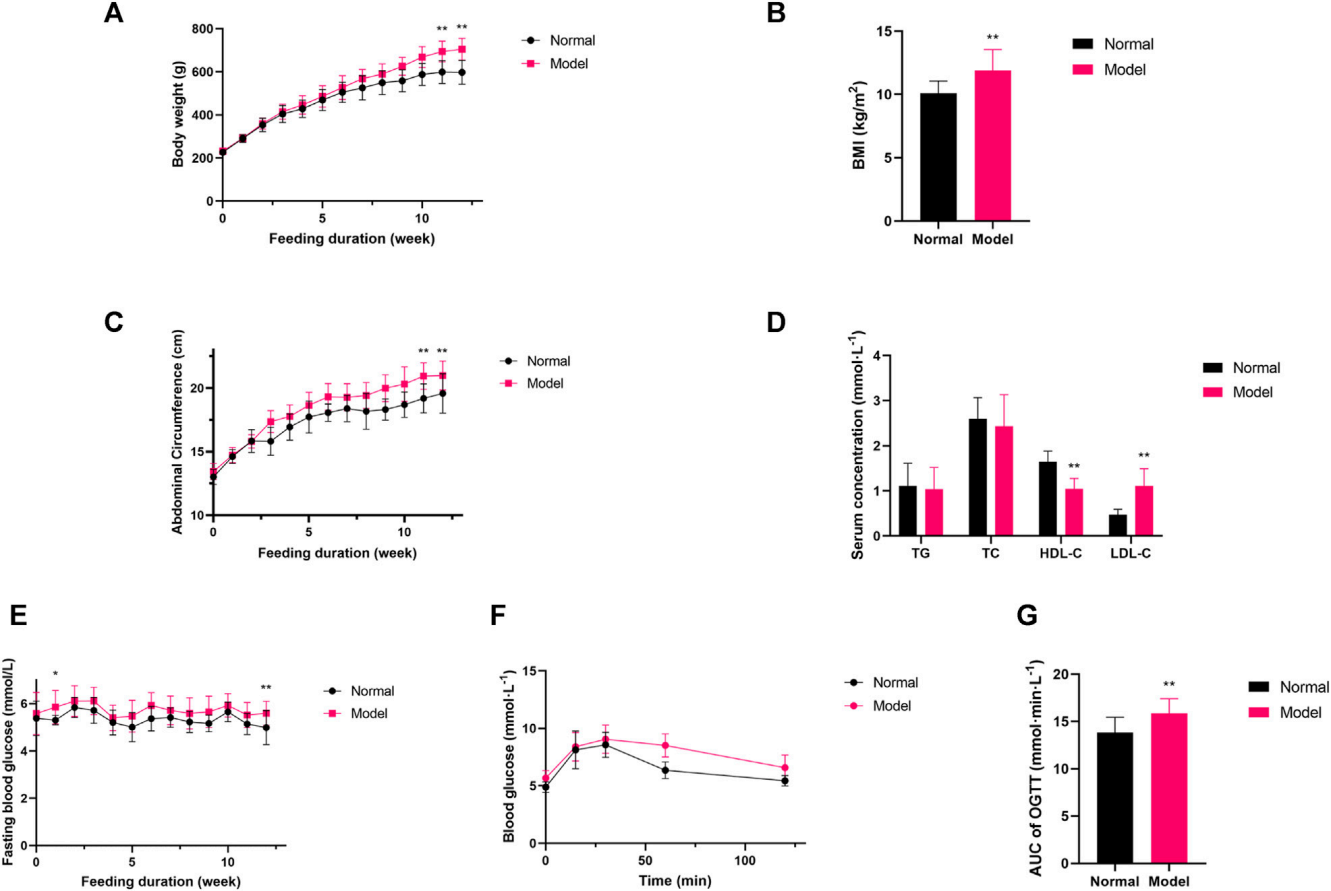
**(A–C)** Variations in body weight, abdominal circumference, BMI; **(D)** Variations in lipid profile; **(E–G)** The quantitative analysis for FBG, AUC of OGTT. Data are expressed as the mean ± SD, *n* = 8 in Normal group and *n* = 40 in Model group. ^*^ *p* < 0.05, ^**^ *p* < 0.01, *versus* Normal group.

After 12 weeks of high-fructose, high-fat diet, the body weight, abdominal circumference and body mass index (BMI) in the Model group was higher than that in the Normal group (Figures 1A–C, *p* < 0.01). In addition, the adipose tissue and liver index of the Model group rats was higher than that of the Normal group (Supplementary Table S3). In terms of lipid indices, Model rats exhibited decreased HDL-C and increased LDL-C, whereas no statistical differences were seen between TG and TC and Normal group rats (Figure 1D, *p* < 0.01). FBG level in the Model group was higher than that in the Normal group (Figure 1E, *p* < 0.05). Moreover, the Model group exhibited impaired glucose tolerance in the oral glucose tolerance test compared with the Normal group (Figures 1F,G, *p* < 0.05). Taken together, the Model group rats showed increased body weight, abdominal circumference and BMI, higher adipose tissue and liver index, decreased HDL-C, and increased FBG along with impaired glucose tolerance (Supplementary Table S4), so the Model group rats were assessed to be consistent with the MetS model.

### 3.2 XSJ reduces fat accumulation in MetS rats

**FIGURE 2:**
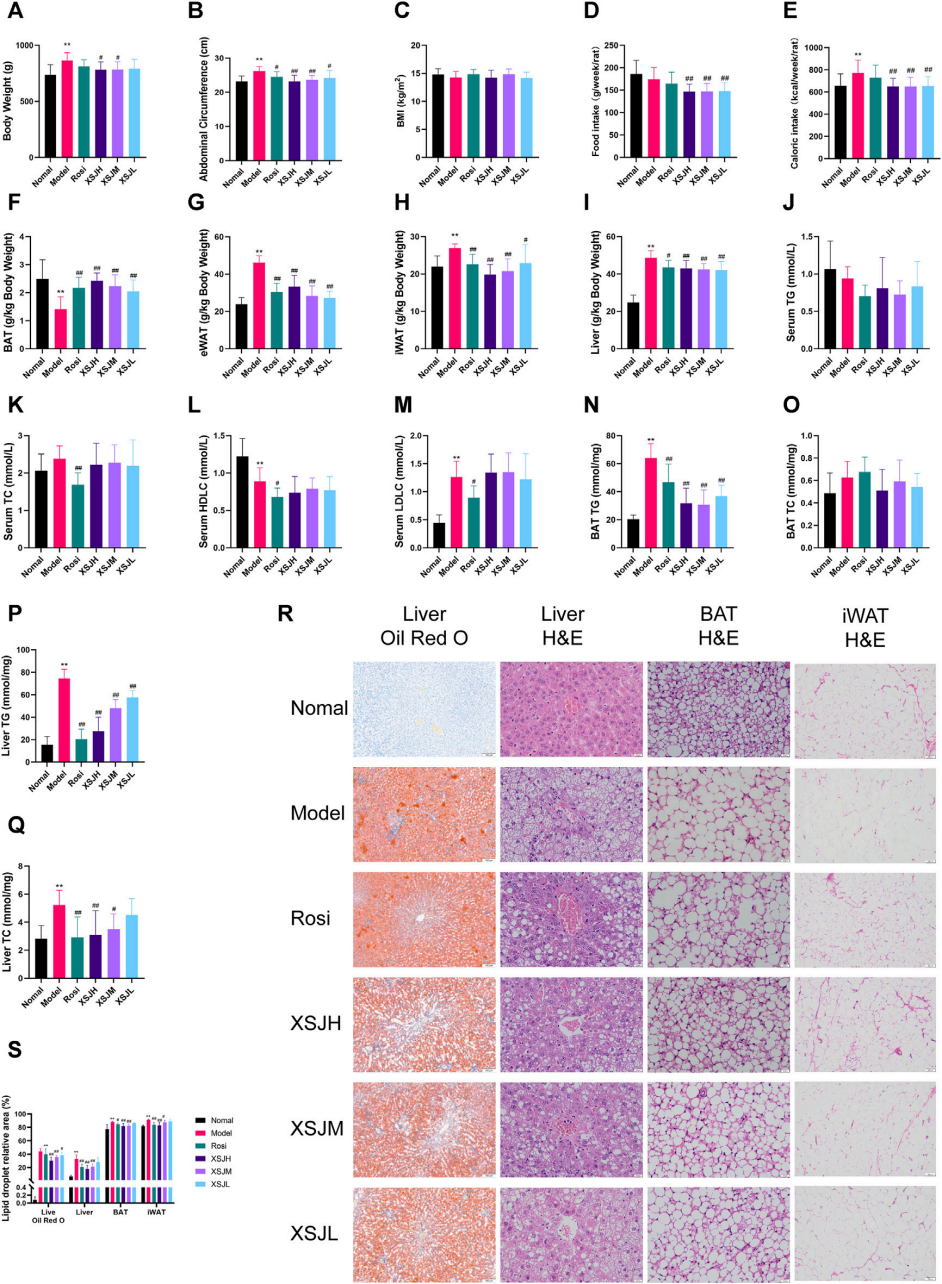
**(A–E)** Variations in body weight, abdominal circumference, BMI, food intake and caloric intake; **(F–I)** Variations in liver, BAT, and iWAT coefficient; **(J–M)** The quantitative analysis for serum TG, TC, HDLC, LDLC; **(N–Q)** The quantitative analysis for TG, TC in BAT and liver. **(R,S)** Liver slices (Oil Red O and H&E) (×100, 100 μm), BAT tissue slices (H&E) (×400, 20 μm) and iWAT (H&E) (×100, 100 μm) photographed under a microscope, and the lipid droplet relative area of liver, BAT and iWAT. Data are expressed as the mean ± SD, *n* = 8 per group. ^*^ *p* < 0.05, ^**^ *p* < 0.01, *versus* Normal group; ^#^ *p* < 0.05, ^##^ *p* < 0.01 *versus* Model group.

Model rats lost weight after 16 weeks of XSJ administration (Figure 2A, *p* < 0.05). The Model group rats’ abdominal circumference was significantly larger than the Normal group, whereas XSJ was able to reduce the abdominal circumference (Figure 2B, *p* < 0.01). There was no significant difference in BMI between the Model group rats and the Normal group rats (Figure 2C, *p* > 0.05). XSJ also reduced the calorie intake compared with the Model group rats (Figures 2D,E, *p* < 0.01). And rats in the Model group showed a decrease in BAT index and an increase in eWAT, iWAT and liver index, whereas XSJ was able to increase BAT index and decrease eWAT, iWAT and liver index (Figures 2F–I, *p* < 0.01). There was a significant increase in BAT TG and liver TG, TC levels in the Model rats compared with the Normal group. Conversely, XSJ was able to improve BAT TG and liver TG, TC levels ((Figure 2J–Q, *p* < 0.01). In addition, adipocytes in the BAT of the Model group showed the morphology of large lipid droplets in a single compartment in H&E staining, and adipocytes were reduced after XSJ administration. Furthermore, based on H&E and Oil Red O staining analysis of the liver, XSJ treatment reduced the number of lipid droplets in the liver and the degree of liver tissue lesions compared to the Model group (Figures 2R,S, *p* < 0.05). These results indicate that XSJ can effectively reduce fat accumulation in Model rats.

### 3.3 XSJ improves insulin resistance, GLP-1 levels and glucose and lipid metabolism disorders in MetS rats by enhancing lipolysis and *β*-oxidation in BAT

**FIGURE 3:**
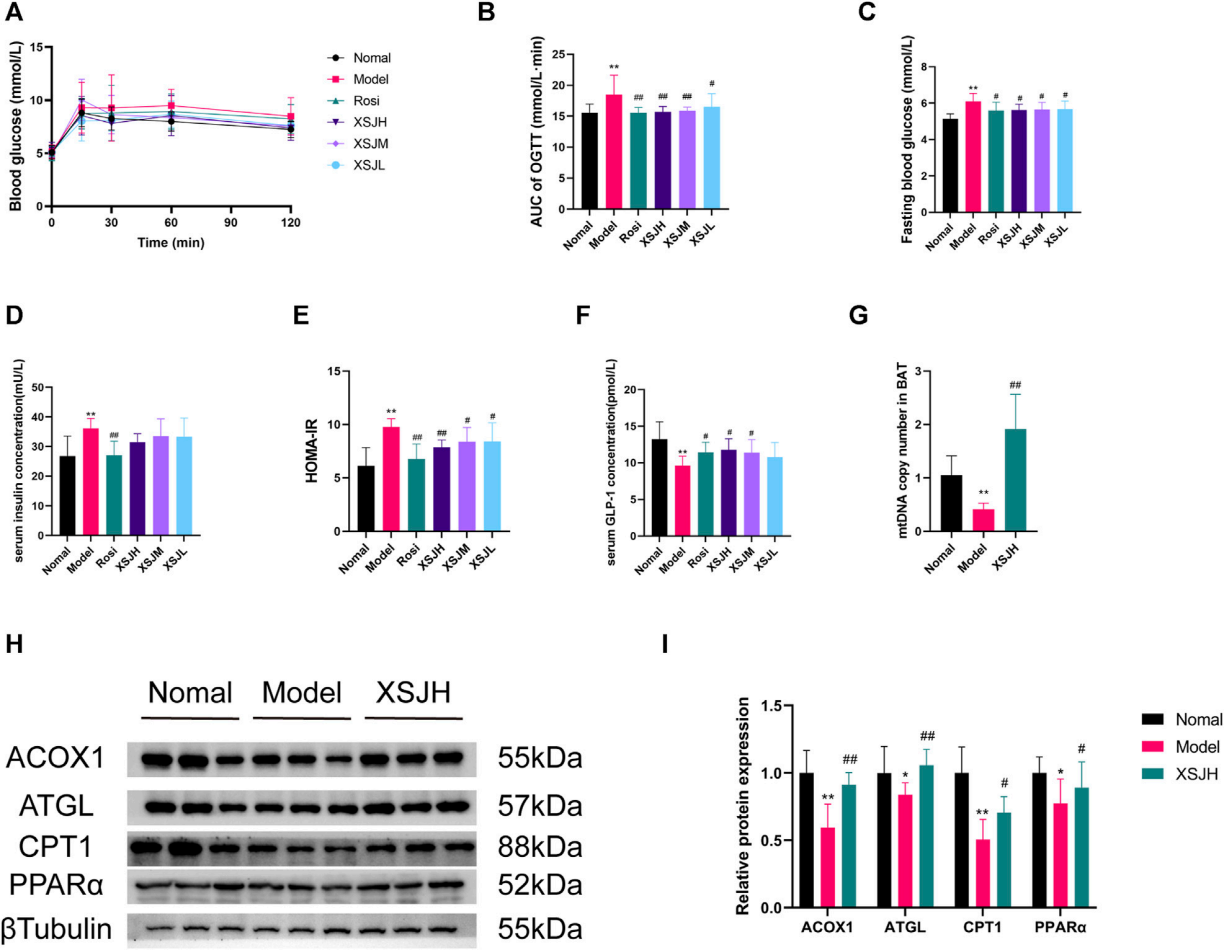
**(A–F)** The quantitative analysis for OGTT, FBG, insulin levels, HOMA-IR and GLP-1 levels; **(G)** The mtDNA copy number in BAT; **(H,I)** Western blotting bands of ACOX1, ATGL, CPT1 and PPAR*α* in BAT tissues and quantitative analysis. Data are expressed as the mean ± SD, *n* = 3–8 per group. ^*^ *p* < 0.05, ^**^ *p* < 0.01, *versus* Normal group; ^#^ *p* < 0.05, ^##^ *p* < 0.01 *versus* Model group.

The rats in the Model group showed elevated fasting blood glucose (FBG) and impaired glucose tolerance, while XSJ was able to improve FBG and glucose tolerance in the rats in the Model group (Figures 3A–E, *p* < 0.01). The results of the insulin resistance index showed that the HOMA-IR in the Model group was significantly higher than that in the Normal group, whereas XSJ could significantly reduce the HOMA-IR (Figure 3E, *p* < 0.01). Moreover, XSJ could significantly increase the serum GLP-1 concentration (Figure 3F, *p* < 0.05). Our findings show that XSJ benefits insulin resistance in Model rats.

Based on the attenuation of fat accumulation in BAT and liver and the improvement of insulin resistance, we investigated the mtDNA copy number and the expression of lipid *β*-oxidation proteins in BAT, including PPAR*α*, ACOX1, CPT1, and lipolysis proteins such as ATGL in BAT. The findings revealed that the mtDNA copy number was enhanced in BAT after XSJ treatment (Figure 3G, *p* < 0.01). Additionally, XSJ increased mtDNA copy number in iWAT compared to the Model group (Supplementary Figure S1, *p* < 0.01). Both PPAR*α*, ACOX1, CPT1, and ATGL were downregulated in the Model rats, whereas XSJ upregulated their expression (Figures 3H,I, *p* < 0.05). These results indicate that XSJ can promote lipolysis and *β*-oxidation in BAT to ameliorate metabolism disorders by regulating the expression of PPAR*α*, ACOX1, CPT1 and ATGL.

### 3.4 XSJ upregulates tyrosine metabolism and noradrenaline-mediated energy metabolism protein expression in BAT

**FIGURE 4:**
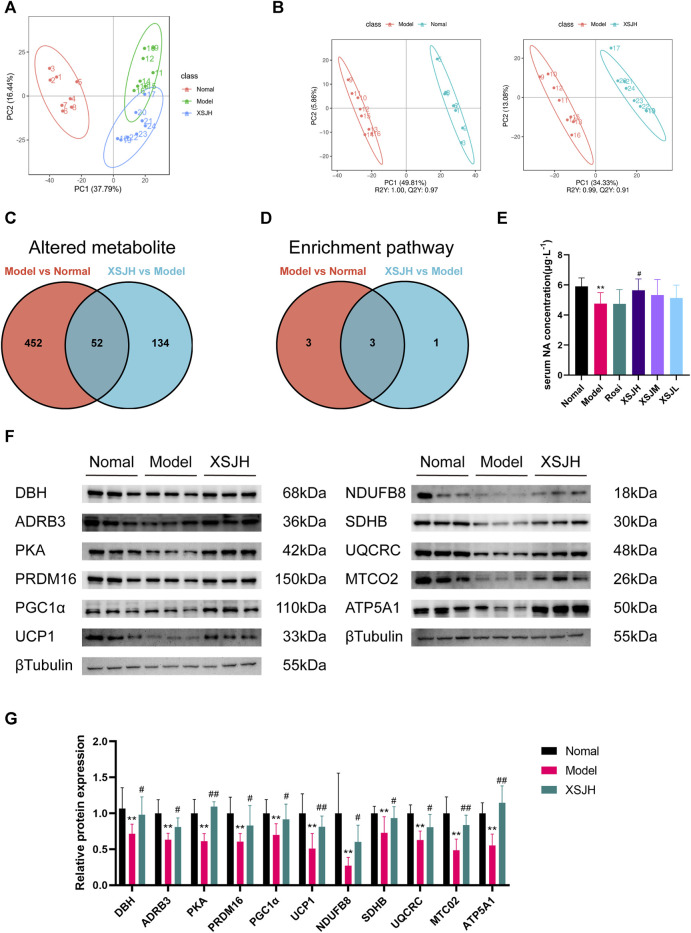
**(A)** PCA among in Normal, Model and XSJH groups; **(B)** OPLS-DA in the Model vs. Normal group and XSJH vs. Model group; **(C)** Differential metabolites among Normal group, Model group and XSJH group; **(D)** Differential KEGG enrichment pathways among Normal group, Model group and XSJH group; **(E)** The analysis of serum NA concentration; **(F,G)** Western blotting bands of DBH, ADRB3, PKA, thermogenic proteins and OXPHOS proteins in BAT tissues and quantitative analysis. Data are expressed as the mean ± SD, *n* = 3–8 per group. ^*^ *p* < 0.05, ^**^ *p* < 0.01, *versus* Normal group; ^#^ *p* < 0.05, ^##^ *p* < 0.01 *versus* Model group.

XSJ exhibited a beneficial influence on the stimulation of BAT, prompting us to delve deeper into the underlying mechanism by which XSJ fosters metabolism through metabolite alterations. The metabolomics data indicated clear separation between the Normal and Model groups in both PCA and PLS-DA plots. The XSJH group exhibited a gradual separation from the Model group, indicating its ability to regulate the metabolites of the rats in the Model group, ultimately leading to an improvement in MetS. (Figures 4A,B).

Differential metabolite screening was performed according to |log2FC|≥1.5, VIP≥1, *p* < 0.05, as shown in Figure 4C where there was a total of 504 altered metabolites between the Normal group and the Model group, and 186 altered metabolites between the XSJH group and the Model group, with a total of 52 shared altered metabolites (Supplementary Table S5). The altered metabolites were analyzed separately for KEGG pathway enrichment using the MBRole 2.0 database. There are 6 enrichment pathways between the Model group and the Normal group, and 4 differential metabolite enrichment pathways between the XSJH group and the Model group, and there are 3 overlapping pathways between them, which were metabolic pathway, phenylalanine, tyrosine and tryptophan biosynthesis pathway, and tyrosine metabolism pathway (Figure 4D). The details of the KEGG pathway enrichment results are listed in Supplementary Tables S6, S7. Among them, tyrosine metabolism pathway is part of the phenylalanine, tyrosine and tryptophan biosynthesis pathway, suggesting that the tyrosine metabolism pathway may be the key pathway for the treatment of MetS by XSJ.

Based on the altered metabolites in tyrosine metabolism, the concentration of noradrenaline (NA) in the colonic contents of rats in the Model group was significantly decreased compared with that of rats in the Normal group, whereas XSJH was able to restore the concentration of NA. So, we examined the concentration of NA in rat serum and found that serum NA concentration in Model rats was significantly decreased compared with rats in the Normal group, whereas XSJ was able to increase serum NA concentration after administration of medium and high doses of XSJ (Figure 4E, *p* < 0.05). The results showed that the expression of NA synthesis and effector proteins (DBH, ADRB3, PKA), thermogenic proteins (UCP1, PGC1*α*, PRDM16), OXPHOS proteins (NDUFB8, SDHB, UQCRC2, MTCO2, and ATP5A1) in BAT in the Model group was decreased, and XSJ could upregulated the expression of the above-mentioned proteins (Figures 4F,G, *p* < 0.05). Although the concentration of NA levels rose, the blood pressure in XSJ group rats was lower than that in Model group rats (Supplementary Figure S2, *p* < 0.05). Combined with previous results that XSJ promotes BAT lipolysis and *β*-oxidation, our study suggests that XSJ accelerates NA synthesis, and therefore the consumption of excess glucose and lipids in the form of thermogenesis through OXPHOS to reduce lipid accumulation.

### 3.5 XSJ treatment changes the gut microbiota composition and strengthens intestinal tight junctions

**FIGURE 5:**
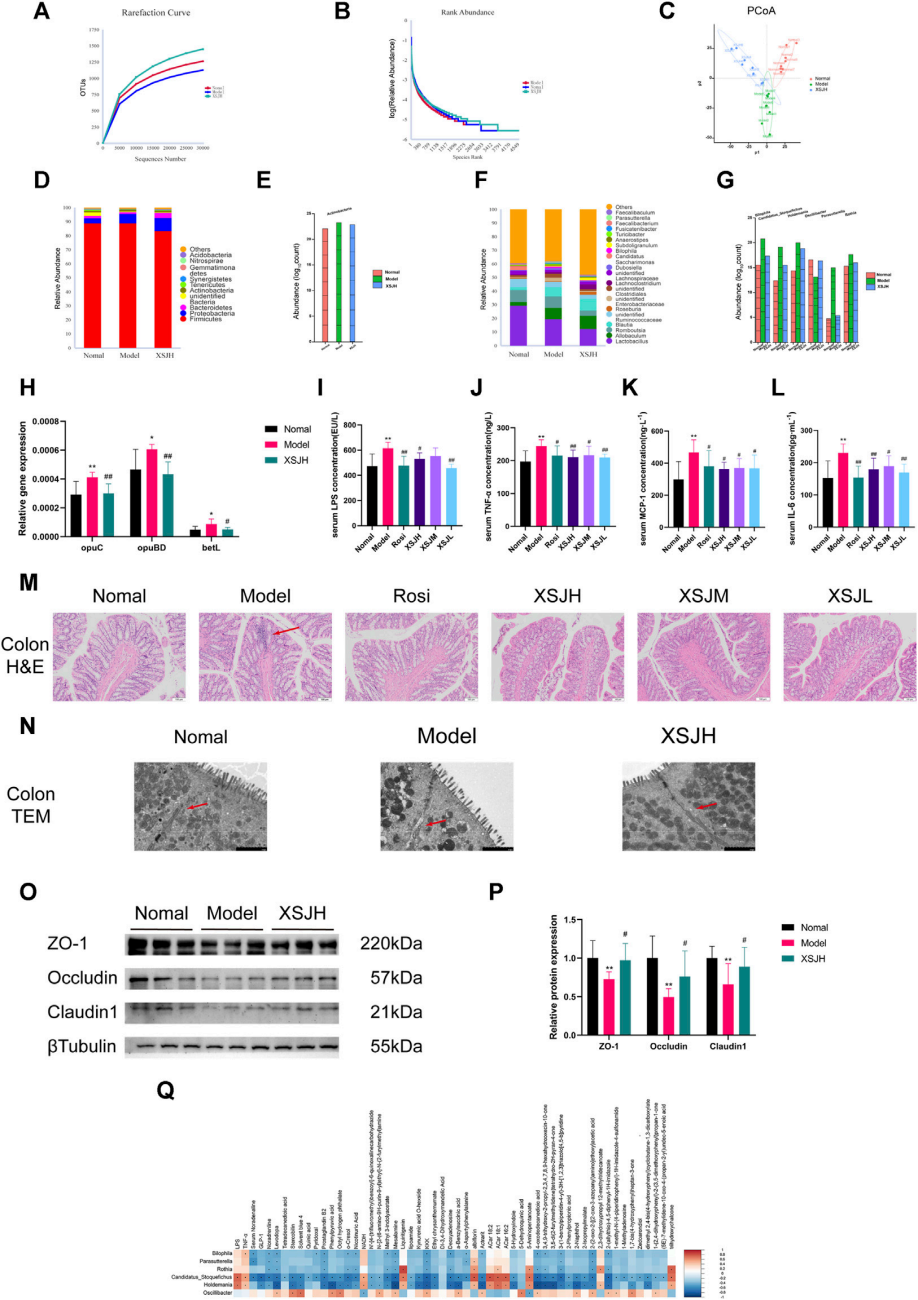
**(A,B)** The rarefaction curve and the rank abundance curve; **(C)** PCoA score; **(D,E)** Relative abundance of dominant and differential microbiota at the phylum level; **(F,G)** Relative abundance of dominant and differential microbiota at the genus level; **(H)** The quantitative analysis for the expression of opuC, opuBD and betL; **(I–L)** The quantitative analysis for LPS, TNF-*α*, MCP-1 and IL-6; **(M)** Colonic epithelial tissue slices (H&E) photographed under a microscope (×100, 100 μm), red arrow points to inflammatory infiltration and glandular atrophy; **(N)** Tight junction of colonic intestinal epithelial cells by TEM (×7000, 2 μm), red arrow points to tight junction; **(O,P)** Western blotting bands of TJ proteins expression in colonic tissues and quantitative analysis. **(Q)** Correlation analysis of differential genera with differential metabolites and serum cytokine. *n* = 8 per group. Data are expressed as the mean ± SD, *n* = 3–8 per group. ^*^ *p* < 0.05, ^**^ *p* < 0.01, *versus* Nomal group; ^#^ *p* < 0.05, ^##^ *p* < 0.01 *versus* Model group. In the correlation analysis, * represents *p* < 0.05, orange indicates a positive correlation, blue indicates a negative correlation and the shade indicates the degree of correlation.

The rarefaction curve can reflect the diversity of microbial communities, the rank abundance curve can explain the species richness and community evenness, and PCoA (principal co-ordinates analysis) can evaluate the separate clustering of the gut microbiota from the different treatment groups. The rarefaction curves flattened, and the rank abundance curve had a larger horizontal axis range and a smoother curve indicating that the sequencing results of this experiment could truly reflect the bacterial abundance in each sample (Figures 5A,B). Based on the weighted unifrac method, the PCoA result showed that the Normal group was significantly separated from the Model group. However, the cluster distance of the XSJH group converged to the Normal group (Figure 5C).

The dominant phyla were mainly classified into *Firmicutes*, *Proteobacteria*, *Bacteroidetes*, *unidentified Bacteria* and *Actinobacteria*. XSJH reduced the abundance of *Actinobacteria* (Figures 5D,E). And the dominant genera were mainly categorized into *Lactobacillus*, *Allobaculum*, *Romboutsia*, *Blautia* and *unidentified Ruminococcaceae*. XSJH upregulated the abundance of *Oscillibacter* and downregulated the abundance of *Bilophila*, *Candidatus Stoquefichus*, *Holdemania*, *Parasutterella* and *Rothia* (Figures 5F,G). The above results suggested that XSJ improved the composition of the gut microbiota in MetS. However, the result of LEfSe (LDA effect size) analysis of the Tax4fun2 pathway prediction also showed that the gut microbiota after XSJ administration was associated with upregulated expression of amino acid metabolism pathway, phenylalanine, tyrosine and tryptophan biosynthesis pathway, and PPAR signaling pathway (Supplementary Figure S3). In terms of functional prediction, as shown in Figure 5H, the microbiota gene of Model rats involved osmoprotectant transport system substrate-binding protein (opuC), osmoprotectant transport system permease protein (opuBD) and glycine betaine transporter (betL) were upregulated (*p* < 0.05), suggesting an altered intestinal osmolality in the Model rats. XSJ restored the expression of osmoprotectant transport gene.

Intestinal barrier leakage may occur with an increase in the abundance of colitis-associated microbiota. As shown in Figures 5I–L, the Model group rats had significantly higher LPS, TNF-*α*, MCP-1 and IL-6, compared to the rats in the Normal group (*p* < 0.05). Through H&E staining analysis, the mucosal intrinsic glands in the colon villi in the Model group were loosely arranged with atrophy, while the intrinsic glands in the colon tissue of the XSJ group were intact or with mild atrophy (Figure 5M). Colon intrinsic glands atrophy indicated that intestinal barrier leakage happened in Model rats. So, we used transmission electron microscopy (TEM) to observe the colonic epithelial tight junctions (TJs). Compared to the Normal group, the TJs of the colonic epithelial cells in the Model group were disrupted and the cellular gaps were enlarged, whereas XSJH was able to restore cellular TJs (Figure 5N). The expression of the TJ protein ZO-1, Occludin and Claudin1 was significantly decreased in the Model group, whereas the expression of TJ proteins was elevated after XSJH administration (Figures 5O, P, *p* < 0.05). The above results demonstrate that XSJ can protect the intestinal barrier to reduce low-grade inflammation due to LPS leakage.

Additionally, *Bilophila*, *Candidatus Stoquefichus*, *Holdemania*, *Parasutterella* and *Rothia* showed a negative correlation with most metabolites, including NA (Figure 5Q). In contrast, *Oscillibacter* had a positive correlation with NA. LPS, TNF-*α*, MCP-1 and IL-6 were positively correlated with *Candidatus Stoquefichus* and *Holdemania*. So, through our analysis, it is suggested that high-fructose, high-fat diet may lead to a disruption of the intestinal environment and an increase in LPS levels, resulting in intestinal barrier leakage and low-grade inflammation.

## 4 Discussion

Metabolic syndrome (MetS) represents a global health challenge, with approximately one-third of US adults affected by this condition (Saklayen, 2018). Similarly, in mainland China, the prevalence of MetS is approximately 24.5% (Li et al., 2016). This escalating prevalence underscores the pressing need for the development of effective and safe therapeutic interventions. Given the bioactive constituents of Xiasangju (XSJ), we posit that XSJ may hold promise as a therapeutic agent for MetS.

Following a 12-week modeling period, rats in the Model group exhibited an increase in body weight, abdominal circumference, and BMI. Concurrently, there was a decline in HDL-C levels, accompanied by an elevation in fasting blood glucose levels and impaired glucose tolerance. Moreover, the adipose tissue and liver index of the Model group rat were larger than those of the Normal group. These findings indicated that a high-fructose, high-fat diet can induce MetS, which is similar to the findings of previous studies (Bae et al., 2022; Choi et al., 2022; Y. Wang et al., 2019). Our 16-week treatment with XSJ extract resulted in positive outcomes, especially high-dose XSJ, including weight loss, reduced abdominal circumference, decreased fat deposition, and improved glucose tolerance. These results suggested that XSJ extract has a therapeutic effect for alleviating MetS.

Normally, excess lipids are absorbed by adipose tissue, preventing ectopic deposition of lipids. When adipose tissue is unable to store lipids, excess lipids accumulate in the liver, leading to lipotoxicity (Sekizkardes et al., 2020). Fat metabolism is altered in non-alcoholic fatty liver disease (NAFLD) due to dysfunction in adipocytes (Lee et al., 2023). Brown adipocytes contain a high mitochondrial density and UCP1 levels and this results in more efficient metabolism capacity (Aquilano et al., 2023). Activated BAT takes up fatty acids and glucose from the bloodstream and releases stored triacylglycerols (Morigny et al., 2021). This has been reported to reduce hepatic lipid deposition and alleviate hepatic steatosis through the consumption of large amounts of lipids, therefore promoting lipid transfer to adipose tissue (Ahmed et al., 2021). Thus, activation of BAT provides a significant reference in the treatment of metabolic disease (Cheng et al., 2021).In this study, XSJ relieved BAT lipid droplet expansion and hepatic steatosis. The activation of BAT by XSJ was indicated by the increased mtDNA copy number and expression of lipolysis and *β*-oxidation proteins in BAT. This improved glucose and lipid metabolism disorder to prevent IR and lipotoxicity.

Tyrosine metabolism pathway is part of the phenylalanine, tyrosine and tryptophan biosynthesis pathway. Catecholamine biosynthesis is an integral module within the tyrosine metabolic pathway. Tyrosine undergoes conversion to noradrenaline (NA) mediated by tyrosine-3-enyl monooxygenase (TH), dopa decarboxylase (DDC), and dopamine beta-hydroxylase (DBH) sequentially. NA is known for its role in promoting energy metabolism. It can activate *β*-adrenergic receptors (*β*-ARs), especially the *β*3-adrenergic receptor (ADRB3) in BAT (Cero et al., 2021). Once NA binds to ADRB3, it stimulates PKA and subsequently lipolysis and *β*-oxidation, resulting in BAT activation (F. Shi et al., 2023). BAT activation can utilize large amounts of lipids and glucose via thermogenesis. This process is indicated by upregulating the expression of thermogenic proteins, including UCP1, PGC1*α* and PRDM16. UCP1 is a specialized protein in adipocytes that dissipates the proton gradient to uncoupled respiration and heat generation. And this is achieved through enhanced OXPHOS by triggering the expression of respiratory chain complexes I, II, III, IV, and V, which are composed of NDUFB8, SDHB, UQCRC2, MTCO2, and ATP5A1. It is important to sustain OXPHOS to keep BAT active and promote high energy expenditure. Recently, an elegant study found that adipocyte thermogenesis is based on the release of NA by sympathetic neurons (Y.-N. Wang et al., 2021). Moreover, the pattern of action of NA neurons depends on the expression of TH and DBH (Zlatkovic et al., 2023). Our metabolomics result indicated XSJ regulated tyrosine metabolism to activate NA biosynthesis to improve MetS. XSJ increased NA levels, with the upregulated expression of NA synthesis and NA-mediated proteins such as DBH, lipolysis proteins, *β*-oxidation proteins, thermogenic proteins, and OXPHOS proteins. This result may suggest that XSJ enhances NA synthesis by activating BAT sympathetic neurons, leading to increased utilization of excess glucose and lipids through thermogenesis via OXPHOS, consequently reducing lipid accumulation. This effect is similar to that observed with the ADRB3 agonist mirabegron in MetS treatment (Finlin et al., 2020). Rats in the Model group exhibited elevated blood pressure and decreased NA concentration, which was reversed in rats in the XSJ group. Recent studies have found that hypertension is not solely caused by elevated levels of NA. Inflammatory states, hyperinsulinemia, and/or obesity may also be contributing factors (Fu et al., 2024; Grassi et al., 2019; H; Wang et al., 2020). So, further studies are needed to investigate the detailed mechanism of XSJ agonist NA biosynthesis from sympathetic nerves regarding the amelioration of MetS.

Growing evidence highlights the substantial influence of gut microbiota composition on systemic metabolism by modulating host metabolites. High-fat diets cause inflammation by gut microbiota leading to metabolic dysregulation. Additionally, fecal microbiota transplantation may transmit metabolic dysfunction (An et al., 2023). The gut microbiota is emerging as a promising avenue for research. Recent investigations have extended our understanding of the gut microbiota’s association with NA levels. Furthermore, gut microbiota-NA crosstalk underpins energetics and thermogenesis under low-temperature conditions (Bo et al., 2019), and gut microbiota-derived compounds increase NA turnover in adipose tissue to enhance energy expenditure (Kim et al., 2017). Conversely, above studies in animal models have revealed increased inflammation alongside reduced NA levels. Thus, the gut microbiota-adipose tissue axis has gained prominence as a regulator of energy metabolism. Here we found a negative correlation between *Bilophila*, *Candidatus Stoquefichus*, *Holdemania*, *Parasutterella* and *Rothia* and NA levels, suggesting their potential roles in energy metabolism dysregulation. Tax4Fun2 pathway prediction also validated that gut microbiota changes the energy metabolism by modulating the transduction of Phenylalanine, tyrosine and tryptophan biosynthesis and PPAR signaling pathway. Phenylalanine, tyrosine and tryptophan biosynthesis may play a key role in XSJ alleviating MetS through tyrosine metabolism leading to the NA biosynthesis. we will explore the mechanism of the AAA biosynthesis to regulate energy metabolism by targeted metabolomic analysis in further study.

*Candidatus Stoquefichus* and *Holdemania* both belong to the *Erysipelotrichaceae* family, have been associated with heightened intestinal inflammation. Existing research highlights a plausible link between *Holdemania* and glucose and lipid metabolism disorders (Zhong et al., 2022; Lippert et al., 2017; T; Zhao et al., 2021; Biassoni et al., 2020). The study indicates that fructose-induced colitis was accompanied by increased *Holdemania* abundance, concomitant with s suppressed ZO-1 expression and increased LPS levels (Song et al., 2023). In contrast, healthier dietary interventions have been associated with decreased *Holdemania* abundance (Barrett et al., 2018; Bjørkhaug et al., 2019). An increased *Candidatus_Stoquefichus* abundance has been noted in colitis, with chronic inflammation and LPS elevation (Feng et al., 2022). Additionally, *Bilophila* and *Parasutterella* have been linked to colitis, inflammation and metabolic dysfunction (Lv et al., 2023; Zahavi et al., 2023; J; Zhao et al., 2023). The expression of tight junction proteins such as ZO-1, Occludin and Claudin1, resulting in increased intestinal barrier permeability and elevated blood LPS levels. LPS can activate the TLR4/MyD88/NF-*κ*B pathway to trigger TNF-*α*, MCP-1 and IL-6 release (L. Shi et al., 2022; Zhu et al., 2023). The increasing serum LPS, TNF-*α*, MCP-1 and IL-6 levels mean the low-grade systemic inflammation in MetS rats. Meanwhile, Elevated LPS promotes TNF-*α* expression and thus inhibits ADRB3 expression in adipocyte (Berkowitz et al., 1998). Also, MCP-1 may cause inflammation to disrupt OXPHOS in adipose tissue (Hiraike et al., 2023). So, low-grade systemic inflammation caused by intestinal barrier leakage and intestinal dysbiosis can impair the function of BAT. In summary, a high-fat, high-fructose diet can cause dysfunction in gut microbiota composition, leading to intestinal barrier leakage, increased levels of LPS, and an inflammatory response. Our results also indicate that Model group rats with intestinal barrier damage exhibit increased serum LPS, TNF-*α*, MCP-1 and IL-6 levels. Also, the osmoprotectant transport system-related gene expression was upregulated, which indicated that the intestinal environment changes in MetS rats. Considering the findings from other studies, *Candidatus Stoquefichus*, *Holdemania*, *Bilophila* and *Parasutterella* may contribute to intestinal barrier disruption, elevated LPS levels and thus body low-grade inflammation. With intestinal barrier permeability and low-grade inflammation increasing, this disorder may potentially impede the activation of BAT driven by NA. Furthermore, there is a need to investigate the correlation between intestinal barrier disruption and host metabolism, and to validate the role of *Candidatus Stoquefichus*, *Holdemania*, *Bilophila* and *Parasutterella*.

Thus, our findings propose that XSJ may alleviate MetS through multiple mechanisms. These potential mechanisms include reinforcing the intestinal barrier integrity to reduce inflammation, regulating the abundance of *Candidatus Stoquefichus, Holdemania, Bilophila and Parasutterella*, and promoting NA synthesis. These mechanisms collectively influence BAT activation for the alleviation of MetS. Our study has limitations in exploring the connection between NA release and gut microbiota. Additionally, we aim to explain the NA turnover in BAT after XSJ-treated and how XSJ regulates the balance of blood pressure when NA is released. The detailed pharmacological mechanism needs to be investigated further.

## 5 Conclusion

**FIGURE 6:**
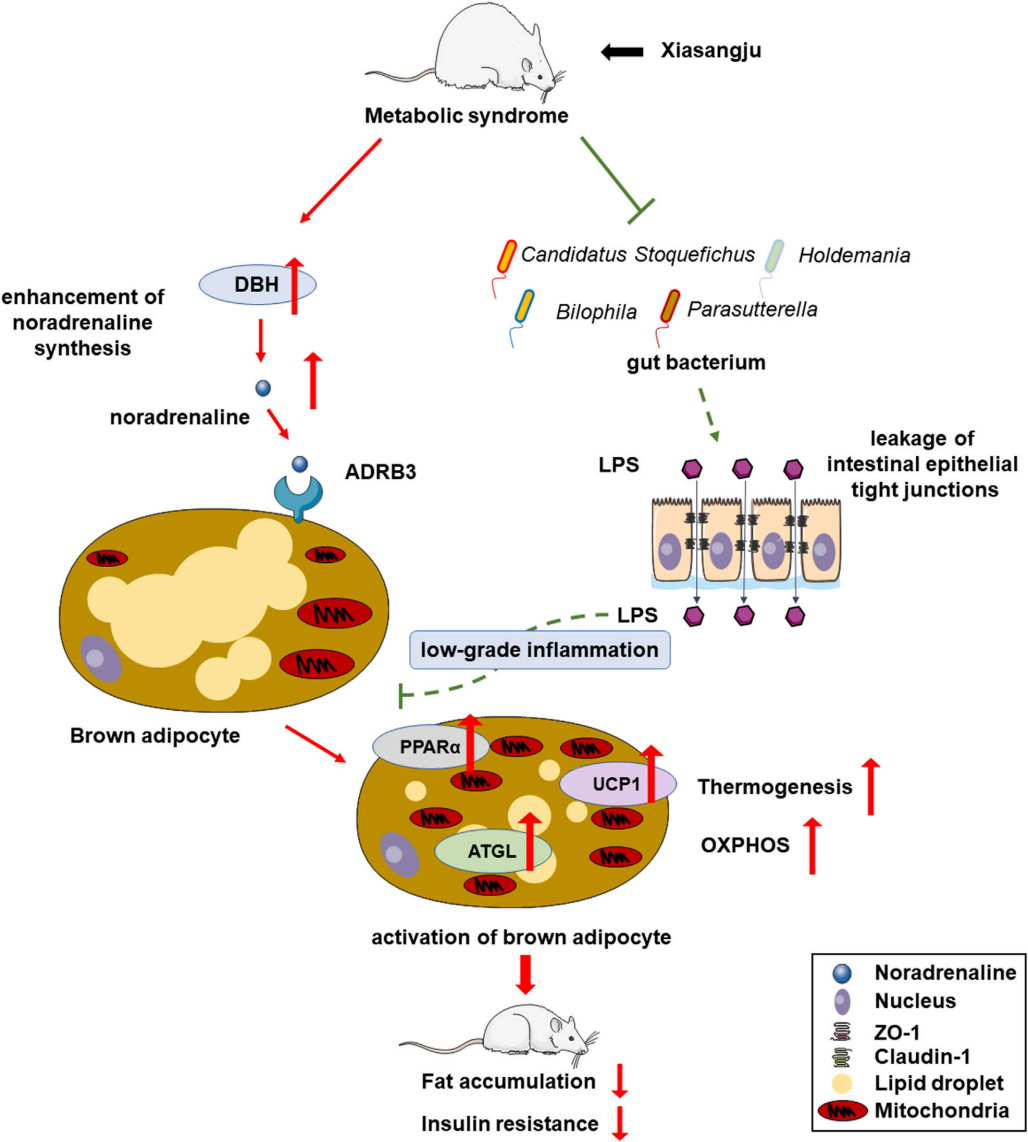
Overview of mechanisms underlying therapeutic effects of Xiasangju extract (XSJ) on metabolic syndrome (MetS).

Our study suggests the potential of XSJ to ameliorate metabolic syndrome by improving energy metabolism and reducing fat deposition. This beneficial effect is attributed to the promotion of NA biosynthesis, activation of brown adipose tissue, fortification of the intestinal barrier, reduce low-grade systemic inflammation (Figure 6). These findings provide valuable insights that may support the use of XSJ extract for the treatment of MetS through NA biosynthesis and NA-mediated energy metabolism modulation.

## Data Availability

The 16S rDNA sequence datasets presented in the study are publicly available. This data can be found here: https://www.ncbi.nlm.nih.gov/sra/PRJNA1085871. The raw data supporting the conclusion of this article will be made available by the authors, without undue reservation.
