# Supplementary material for: Xiasangju alleviate metabolic syndrome by enhancing noradrenaline biosynthesis and activating brown adipose tissue
Source: Front Pharmacol. 2024 Mar 21;15:1371929. doi: 10.3389/fphar.2024.1371929 (PMC10993144; doi:10.3389/fphar.2024.1371929)
Supplement: Supplementary file 1 [file DataSheet1.pdf]

Supplementary Material

1 Supplementary Figures and Tables

1.1 Supplementary Figures

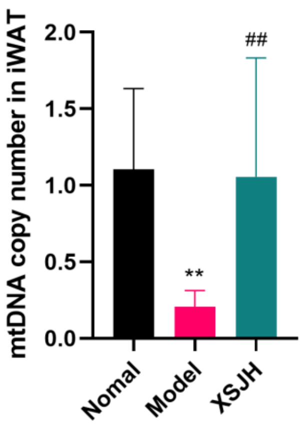

Supplementary Figure 1. The mtDNA copy number in iWAT. Data are expressed as the mean ± SD, n = 3 per group. $^{*}p < 0.05$, $^{**}p < 0.01$, versus Normal group; $^{\#}p < 0.05$, $^{\#\#}p < 0.01$ versus Model group.

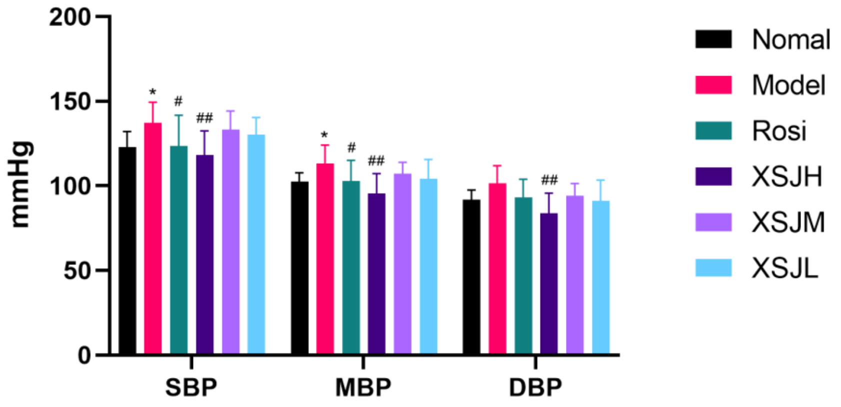

Supplementary Figure 2. Variations in blood pressure. Data are expressed as the mean ± SD, n = 8 per group. $^{*}p < 0.05$, $^{**}p < 0.01$, versus Normal group; $^{\#}p < 0.05$, $^{\#\#}p < 0.01$ versus Model group.

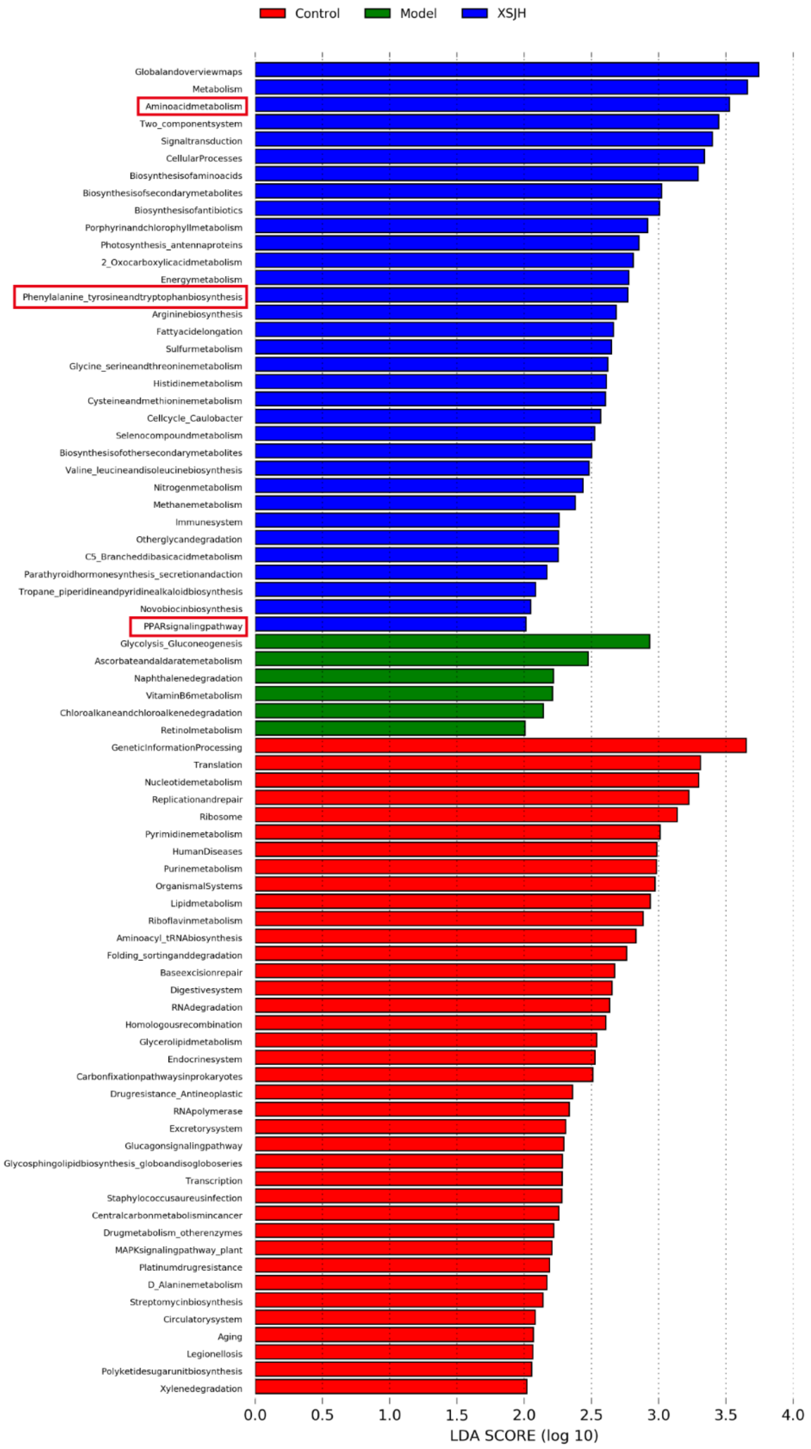

Supplementary Figure 3. LEfSe of Tax4fun2 pathway prediction, the diagram labels red squares with pathways related to noradrenaline biosynthesis and lipid metabolism.

1.2 Supplementary Tables

Supplementary Table 1. Diet formulation

Standard chow diet SWS9102		XT301-1(Adjusted from Research Diets, D09100303)	
Standard chow diet	100%	Standard chow diet	25.9%
		Carbohydrates	
		Fructose	30%
		Fat	
		Lard	16%
		Sesame oil	3%
		Protein	
		Casein	16%
		Bile salt	0.5%
		Triglyceride	1.5%
		Propylthiouracil	0.1%
		Ash	7%
Calories from carbohydrate	67.4%	Calories from carbohydrate	41.9%
Calories from Fat	12.0%	Calories from Fat	40.1%
Calories from protein	20.6%	Calories from protein	18.0%
Energy Density	3.53 Kcal/g	Energy Density	4.43 Kcal/g

Supplementary Table 2. Primer sequences of rat genes.

Target Gene	Forward Primer (5'-3')	Reverse Primer (5'-3')
Cox2	AAAGCCAGGGGAGCTACGACTATT	CGGCCTGGGATTGCGTCTGTTT
β-actin	ACCCACACTGTGCCCATCTAC	TCGGTGAGGATCTTCATGAGGTA

Supplementary Table 3. The body composition of Normal group and Model group rat after 12 weeks of a high-fructose, high-fat diet

Group	Body weight (g)	Abdominal circumference (cm)	Interscapular BAT (g/kg)	eWAT (g/kg)	iWAT (g/kg)	Liver (g/kg)
Normal	496.6	16.7	2.758	20.217	20.882	25.050
Model	593.6	16.6	2.257	28.607	24.165	39.791

Supplementary Table 4. Main features of Normal group and Model group rats after 12 weeks of a high-fructose, high-fat diet

Group	Body Weight (g)	BMI (kg/m2)	Abdominal circumference (cm)	Fasting blood glucose (mmol/L)	AUC of OGTT (mmol/L·min^{-1})	TG (mmol/L)	TC (mmol/L)	HDLC (mmol/L)	LDLC (mmol/L)
Normal	621.98+70.97	10.16+0.92	20.08+1.85	5.09+0.71	13.84+1.60	1.26+0.55	2.64+0.43	1.68+0.22	0.45+0.12
Model	681.96+63.75	11.84+1.59	20.70+1.19	5.61+0.51	15.77+1.47	0.96+0.47	2.34+0.69	1.01+0.23	1.08+0.38

Supplementary Table 4. Altered metabolites among Normal, Model and XSJH groups

Metabolites	Model vs Nomal				XSJH vs Model			
	VIP	*p*	Log_2FC	Trend	VIP	*p*	Log_2FC	Trend
1-methyl-N-(4-piperidinophenyl)-1H-imidazole-4-sulfonamide	1.394	7.1E-12	-6.397	down	2.184	1.1E-05	7.321	up
1-(2,4-dihydroxyphenyl)-2-(3,5-dimethoxyphenyl)propan-1-one	1.346	1.6E-10	-5.326	down	2.131	2E-05	6.2733	up
3,5-di(2-furylmethylidene)tetrahydro-2H-pyran-4-one	1.635	3.7E-10	-2.865	down	1.283	0.0001	1.621	up
Pyridoxal	1.051	3.8E-10	-1.652	down	2.522	6.7E-10	2.6946	up
alpha-Benzylsuccinic acid	1.67	7.7E-10	-4.405	down	1.518	5.7E-07	2.7181	up
2-Naphthol	1.679	9.6E-10	-4.166	down	1.462	2.7E-07	2.4458	up
Actrarit	1.596	1.1E-09	-2.939	down	1.278	0.00042	1.6898	up
Mesalamine	1.654	1.4E-09	-2.616	down	1.681	4.6E-08	1.7992	up
Phenylpyruvic acid	1.612	6E-09	-5.506	down	1.127	0.00108	3.0145	up
KKK	1.556	1.8E-08	-3.63	down	2.025	2.3E-07	3.145	up
2-{2-oxo-2-[(2-oxo-3-azepanyl)amino]ethoxy}acetic acid	1.379	1.9E-08	-1.711	down	2.196	2.2E-08	1.835	up
2-(allylthio)-4,5-diphenyl-1H-imidazole	1.474	1.5E-07	-2.057	down	1.833	5E-06	1.754	up
Nicotinuric Acid	1.353	2.8E-07	-1.534	down	1.822	0.00027	1.5055	up
1,4-dihydroxy-1,4-dimethyl-7-(propan-2-ylidene)-decahydroazulen-6-one	1.296	3.4E-07	-4.655	down	2.231	1.4E-07	5.6724	up
5-Hydroxyindole	1.611	3.4E-07	-4.274	down	1.298	6.1E-05	2.3871	up
3,8,9-trihydroxy-10-propyl-3,4,5,8,9,10-hexahydro-2H-oxecin-2-one	1.586	3.4E-07	-3.46	down	1.487	4E-05	2.1548	up
Noradrenaline	1.268	3.7E-07	-1.807	down	2.333	1.4E-10	2.1822	up
Dl-3,4-Dihydroxymandelic Acid	1.177	4.9E-07	-2.15	down	1.619	0.00869	2.5353	up
5-Dehydroquinic acid	1.212	5.6E-07	-2.814	down	1.918	0.00061	3.6058	up
o-Cresol	1.414	7.2E-07	-4.496	down	1.766	0.0002	4.2095	up
Prostaglandin B2	1.451	1.2E-06	-2.391	down	1.836	8.1E-06	2.0349	up
Tetradecanedioic acid	1.093	1.3E-06	-3.258	down	2.305	5.5E-07	4.8435	up
Levodopa	1.546	2.5E-06	-2.064	down	1.729	2.6E-06	1.5449	up
1,3-dimethyl 2,4-bis(4-hydroxyphenyl)cyclobutane-1,3-dicarboxylate	1.526	2.8E-06	-3.416	down	1.129	0.00113	1.7706	up

alpha-Aspartylphenylalanine	1.271	2.9E-06	-3.212	down	2.296	9.4E-08	4.0277	up
1,7-bis(4-hydroxyphenyl)heptan-3-one	1.557	3.9E-06	-3.614	down	1.2	4.4E-05	1.7795	up
4-oxododecanedioic acid	1.577	4.3E-06	-3.091	down	1.651	4.3E-05	2.1575	up
(5E)-7-methylidene-10-oxo-4-(propan-2-yl)undec-5-enoic acid	1.221	5.1E-06	-4.926	down	2.292	1.3E-06	6.326	up
(2E)-1-(2,4-dihydroxyphenyl)-3-(4-hydroxyphenyl)prop-2-en-1-one	1.459	5.4E-06	4.3764	up	1.238	0.00202	-2.307	down
N'-[4-(trifluoromethyl)benzoyl]-6-quinoxalinecarbohydrazide	1.226	6.5E-06	-2.975	down	2.193	2.7E-06	3.5328	up
Solvent blue 4	1.259	2.7E-05	-2.042	down	1.244	0.02432	1.515	up
5-Aminopentanoate	1.321	2.7E-05	2.656	up	1.396	0.00323	-1.716	down
Octyl hydrogen phthalate	1.225	2.9E-05	-2.367	down	1.167	0.02295	1.839	up
1-Methyladenosine	1.185	3.2E-05	-3.133	down	2.325	7.7E-07	4.3209	up
N-[2-(6-amino-9H-purin-9-yl)ethyl]-N-(2-furylmethyl)amine	1.158	3.8E-05	-3.128	down	1.827	0.00167	3.773	up
Deoxyadenosine	1.39	5.5E-05	-2.035	down	1.727	5.3E-05	1.6921	up
ACar 16:1	1.333	6.9E-05	3.686	up	1.193	0.01743	-1.553	down
2-Isopropylmalate	1.476	7.2E-05	-2.368	down	1.675	1.2E-08	1.6393	up
Stercobilin	1.31	8.1E-05	-2.546	down	1.285	0.01948	1.8742	up
Ethyl chrysanthemumate	1.356	8.7E-05	-1.967	down	1.71	7.5E-06	1.6077	up
3-(1-benzylpiperidin-4-yl)-3H-[1,2,3]triazolo[4,5-b]pyridine	1.361	0.0001	-2.281	down	1.886	0.00024	2.0679	up
Liquiritigenin	1.425	0.00011	4.6886	up	1.09	0.00065	-2.753	down
ACar 18:2	1.186	0.00013	4.12	up	1.233	0.02639	-1.68	down
albiflorin	1.343	0.00018	2.7289	up	1.156	0.01106	-1.609	down
Kynurenic acid O-hexside	1.285	0.00026	-3.499	down	1.428	0.00072	2.5983	up
2-Phenylpropionic acid	1.227	0.00027	-3.168	down	1.34	0.00467	2.2983	up
2,3-dihydroxypropyl 12-methyltridecanoate	1.229	0.00029	2.4718	up	1.265	0.00838	-1.695	down
ACar 18:1	1.242	0.0003	4.0267	up	1.185	0.01898	-1.527	down
lipoamide	1.15	0.00168	-2.367	down	1.849	3.5E-06	2.1269	up
NADH	1.22	0.00376	1.9383	up	1.394	0.00055	-2.2	down
Quinic acid	1.022	0.00465	-1.53	down	1.819	3.8E-05	1.6447	up
Methyl 3-indolyacetate	1.08	0.00868	-1.604	down	1.627	0.00805	1.5784	up

Supplementary Table 5. Altered metabolite KEGG pathway enrichment between Normal and Model groups

Pathway	N	n	M	m	*p*	FDR correction
Metabolic pathways	119	85	3069	1455	<0.001	4.36E-06
Pyrimidine metabolism	119	11	3069	59	<0.001	0.000439
ABC transporters	119	11	3069	90	<0.001	0.01175
Tyrosine metabolism	119	10	3069	76	<0.001	0.01175
Aminoacyl-tRNA biosynthesis	119	9	3069	75	0.002	0.034361
Phenylalanine, tyrosine and tryptophan biosynthesis	119	5	3069	27	0.003	0.044242

Supplementary Table 6. Altered metabolite KEGG pathway enrichment between Model and XSJH groups

Pathway	N	n	M	m	*p*	FDR correction
Phenylalanine, tyrosine and tryptophan biosynthesis	32	5	3069	27	<0.001	0.000159
Tyrosine metabolism	32	5	3069	76	<0.001	0.01266
Metabolic pathways	32	23	3069	1455	0.004	0.036928
